# *Pneumocystis jirovecii* in Patients With Cystic Fibrosis: A Review

**DOI:** 10.3389/fcimb.2020.571253

**Published:** 2020-09-29

**Authors:** Pierre Bonnet, Solène Le Gal, Enrique Calderon, Laurence Delhaes, Dorothée Quinio, Florence Robert-Gangneux, Sophie Ramel, Gilles Nevez

**Affiliations:** ^1^Laboratoire de Parasitologie et Mycologie, Hôpital de La Cavale Blanche, CHU de Brest, Brest, France; ^2^Groupe d'Etude des Interactions Hôte-Pathogène (ER, GEIHP), Université d'Angers, Université de Brest, Brest, France; ^3^CIBER de Epidemiologia y Salud Publica and Instituto de Biomedicina de Sevilla, Hospital Universitario Virgen del Rocio/CSIC/Universidad de Sevilla, Seville, Spain; ^4^Laboratory of Parasitology and Mycology, Bordeaux University Hospital, Bordeaux, France Inserm U1045 – University of Bordeaux, Bordeaux, France; ^5^Univ Rennes, CHU Rennes, Inserm, EHESP, Irset (Institut de Recherche en Santé Environnement Travail), UMR_S 1085, Rennes, France; ^6^Centre de Ressources et de Compétences de la Mucoviscidose, Fondation Ildys, Roscoff, France

**Keywords:** *Pneumocystis jirovecii*, *Pneumocystis* pneumonia, cystic fibrosis, pulmonary colonization, genomic diversity, microbiota, *Pneumocystis* primary infection, lung transplantation

## Abstract

*Pneumocystis* pneumonia (PCP) remains the most frequent AIDS-defining illness in developed countries. This infection also occurs in non-AIDS immunosuppressed patients, e.g., those who have undergone an organ transplantation. Moreover, mild *Pneumocystis jirovecii* infections related to low pulmonary fungal burden, frequently designated as pulmonary colonization, occurs in patients with chronic pulmonary diseases, e.g., cystic fibrosis (CF). Indeed, this autosomal recessive disorder alters mucociliary clearance leading to bacterial and fungal colonization of the airways. This mini-review compiles and discusses available information on *P. jirovecii* and CF. It highlights significant differences in the prevalence of *P. jirovecii* pulmonary colonization in European and Brazilian CF patients. It also describes the microbiota associated with *P. jirovecii* in CF patients colonized by *P. jirovecii*. Furthermore, we have described *P. jirovecii* genomic diversity in colonized CF patients. In addition of pulmonary colonization, it appears that PCP can occur in CF patients specifically after lung transplantation, thus requiring preventive strategies. In other respects, *Pneumocystis* primary infection is a worldwide phenomenon occurring in non-immunosuppressed infants within their first months. The primary infection is mostly asymptomatic but it can also present as a benign self-limiting infection. It probably occurs in the same manner in CF infants. Nonetheless, two cases of severe *Pneumocystis* primary infection mimicking PCP in CF infants have been reported, the genetic disease appearing in these circumstances as a risk factor of PCP while the host-pathogen interaction in older children and adults with pulmonary colonization remains to be clarified.

## Introduction

*Pneumocystis jirovecii* is a transmissible fungus that causes severe pneumonia in immunocompromised patients (Walzer and Cushion, 2005). However, *Pneumocystis* infections cover a spectrum of presentations, in which PCP represents only a small proportion whereas most *Pneumocystis* infections correspond to mild diseases due to low levels of parasitism (Morris and Norris, 2012). Indeed, polymerase chain reaction (PCR) assays have revealed that patients without PCP can be infected with a few organisms (Nevez et al., 1999). In these circumstances, the term “pulmonary colonization with *P. jirovecii*” is frequently used (Morris and Norris, 2012). Pulmonary colonization arises in immunocompromised patients as well as immunocompetent patients with underlying lung diseases. The potential role of the fungus as a morbidity cofactor has been suggested (Morris and Norris, 2012). Moreover, these colonized patients may act as infectious sources (Le Gal et al., 2012, 2015a). For these reasons, the characterization of colonized populations as well as that of *P. jirovecii* organisms in colonized populations are required. For instance, it has been established that patients with cystic fibrosis (CF) can be colonized by *P. jirovecii* (Calderón et al., 2010). CF is due to mutations of the cystic fibrosis transmembrane conductance regulator (CFTR) gene that encodes a chloride channel involved in electrolytic exchanges and is associated with impairment of the mucociliary clearance (Ferec and Cutting, 2012). The resulting thickening of mucus favors pulmonary colonization or infections due to bacteria and fungi (Lipuma, 2010). The first data on pulmonary colonization with *P. jirovecii* in CF patients were obtained in Munich, Germany (Sing et al., 2001). Further studies provided additional data in this field (Respaldiza et al., 2005; Le Gal et al., 2010; Hernandez-Hernandez et al., 2012; Pederiva et al., 2012; Green et al., 2016; Nevez et al., 2018). Moreover, PCP can also occur in CF patients (Royce and Blumberg, 2000; Quattrucci et al., 2005; Solé et al., 2006; Kaur et al., 2016; Wojarski et al., 2018). The objective of this review is to compile available information on *P. jirovecii* and CF worldwide.

## Pulmonary Colonization With *P. JIROVECII* in CF Patients

### Differences of *P. jirovecii* Prevalence in European and Brazilian CF Patients

In Munich, Germany, sputum specimens from 95 patients were prospectively assayed using a nested-PCR amplifying the mitochondrial large subunit rRNA (mtLSUrRNA) gene (Sing et al., 2001). *P. jirovecii* was detected in seven patients (7.4%). Two patients who were submitted to recurrent sampling remained positive for *P. jirovecii* detection over a 4–6-week period.

In Seville, Spain, sputum or oropharyngeal wash specimens from 88 patients were assayed using a nested-PCR amplifying the mtLSUrRNA gene. *P. jirovecii* was detected in 19 patients (21.6%) (Respaldiza et al., 2005).

In Brest, Brittany, France, sputum specimens from 76 patients were retrospectively assayed using both qPCR and nested-PCR amplifying the mtLSUrRNA gene (Le Gal et al., 2010). *P. jirovecii* was detected in one patient (1.3%). A second study was performed in Rennes, the largest city in Brittany, in the course of which sputum specimens from 86 patients were retrospectively assayed using the same qPCR (Nevez et al., 2018). The fungus was detected in three patients (3.5%).

In a French multicenter study, sputum specimens from 104 patients who lived in the following four cities, Angers, Bordeaux, Dunkirk and Lille, were prospectively analyzed using a conventional PCR combined with a qPCR amplifying the mtLSUrRNA gene (Hernandez-Hernandez et al., 2012). The fungus was detected in 13 patients (12.5%).

In Manchester, United Kingdom, sputum specimens from 111 patients were prospectively assayed using a qPCR amplifying the mtLSUrRNA gene (Green et al., 2016). The fungus was detected in nine patients (8.1%) with a higher frequency in specimens from patients presenting with pulmonary exacerbation than in those from patients without exacerbation (9.2 vs. 2%, *p* = 0.03). One patient, submitted to recurrent sampling, remained positive for *P. jirovecii* detection over a 3-month period.

In Porto Alegre, Brazil, BAL specimens from 34 patients were retrospectively assayed using a nested-PCR amplifying the mtLSUrRNA gene. *P. jirovecii* was detected in 13 patients (38.2%) (Pederiva et al., 2012).

The frequencies of *P. jirovecii* colonization in patients enrolled in these seven studies differ significantly. Specifically, the frequency observed in Brittany, Western France, differed from that was observed in Seville, Porto Alegre, and other French regions, whereas it did not differ from that observed in Munich and in Manchester (Sing et al., 2001; Respaldiza et al., 2005; Le Gal et al., 2010; Hernandez-Hernandez et al., 2012; Pederiva et al., 2012; Green et al., 2016; Nevez et al., 2018) (Table 1).

**Table 1:** Comparison of characteristics of cystic fibrosis (CF) patients who underwent *Pneumocystis jirovecii* detection in pulmonary specimens [partly reproduced from Nevez et al. (2018)].

	**Germany (Munich)**	**Spain (Seville)**	**Brittany, France (Rennes and Brest taken together)**	**Other French regions and cities (Pays de la Loire, Angers; South West, Bordeaux; North, Dunkirk and Lille)**	**Brazil (Porto Alegre)**	**United Kingdom (Manchester)**
Number of patients	95	88	162	104	34	111
Median age, years [range]	23.2	15.8 [1–35]	19.0 [3 months-41]	24.0 [15–32]	11.0 [1–35]	31+/– 10 (mean; sd)
Sex ratio M/F	52/43	41/47	81/81	50/54	17/17	59/52
Inclusion period	NA	May 2001-July 2002	February 2005-August 2007	October 2006-March 2009	March 2006 – August 2009	October 2013 – May 2014
Number of specimens	137	88	324	146	NA	226
Pulmonary samples examined for *Pneumocystis jirovecii* detection	Sputa	Sputa (54) and oropharyngeal washes (34)	Sputa	Sputa	BAL	Sputa
Technique of *Pneumocystis jirovecii* detection in pulmonary specimens	Nested PCR targeting mtLSUrRNA gene	Nested PCR targeting mtLSUrRNA gene	Real-time PCR targeting mtLSUrRNA gene	Conventional PCR followed by a real-time PCR targeting mtLSUrRNA gene	Nested PCR targeting mtLSUrRNA gene	Real-time PCR targeting mtLSUrRNA gene
Number of patients with a prior treatment with cotrimoxazole	0	2	NA (Rennes) 30 out of 76 (Brest)	NA	5 (cotrimoxazole or azithromycin in the 6 months preceding sampling)	102
Number of patients colonized with *Pneumocystis jirovecii*	7 (7.4%; CI 95: 2.1–12.7%)	19 (21.6%; CI 95: 12.9–30.1%)	4 (2.5%; CI 95: 0.1–4.9%)	13 (12.5%; CI 95: 6.1–18.9%)	13 (38.2%; CI 95%:21.9–54.5%)	9 (8.1%; CI 95%: 3–13.2%)

*BAL, bronchoalveolar lavage; CI, confidence interval; F, female; M, male; mtLSUrRNA, mitochondrial large subunit ribosomal RNA; NA, not available*.

### Pulmonary Microbiota and *P. jirovecii* Colonization in CF Patients

Pulmonary microbiota characteristics in CF patients with or without *P. jirovecii* colonization were partially described in four studies. In the Brest study, 74 out of 76 patients were colonized by bacteria and/or fungi (Le Gal et al., 2010). The low incidence of *P. jirovecii* (1.3%) was not compatible with any comparison of microbiota in patients colonized by *P. jirovecii* or not. It is noteworthy, that the patient colonized by *P. jirovecii* was also colonized by *Pseudomonas aeruginosa*. In the Rennes study, 86 patients were colonized by bacteria and/or fungi (Nevez et al., 2018). Likewise, the low incidence of *P. jirovecii* (3.5%), rendered any comparison difficult. The three patients colonized by *P. jirovecii* were also colonized by *Staphyloccus aureus, Stenotrophomonas maltophilia, Candida glabrata* (first patient), by *Saccharomyces* sp. and *Haemophilus influenzae* (second patient), by *Aspergillus fumigatus, Candida albicans, P. aeruginosa* (third patient). In the French multicenter study (Hernandez-Hernandez et al., 2012), the presence of *P. aeruginosa* mucoid-strains showed a negative association with *P. jirovecii* presence. In the Manchester study, no difference of microbiota in CF patients with exacerbation and with or without *P. jirovecii* colonization was observed (Green et al., 2016). These studies were based on the results of microbial cultures combined with those of *P. jirovecii* DNA amplification since this fungus is uncultivable. Recently, next generation sequencing (NGS) studies showed that the pulmonary microbiota of CF patients was more complex than initially supposed, considering the results of microbial cultures (Caverly et al., 2019; Cuthbertson et al., 2020; Enaud et al., 2020; Soret et al., 2020). Nonetheless, none of these recent studies has specifically focused on the relationship between microbiota and *P. jirovecii* colonization.

### *P. jirovecii* Genomic Diversity in CF Patients

The pioneer study on *P. jirovecii* genotyping in Seville identified three different mtLSUrRNA genotypes in 11 patients colonized by the fungus (Respaldiza et al., 2005). Genotype 1 (85C/248C) was the most prevalent (5/11, 45.5%) followed by genotype 3 (85T/248C) (3/11, 27.3%) and genotype 2 (85A/248C) (2/11, 18.2%). The genotype distribution was similar to that previously described in a Spanish population presenting PCP and chronic pulmonary diseases, but excluding CF (Montes-Cano et al., 2004).

In the multicenter French study, genotype 1 predominated in the overall population (~60%), consistent with the results of the Spanish study (Respaldiza et al., 2005; Hernandez-Hernandez et al., 2012). However, geographic variations emerged with higher prevalence of genotype 2 in Dunkirk in the absence of genotype 3, whereas genotype 1 predominated in Angers in the absence of genotype 2 (Hernandez-Hernandez et al., 2012). In the other French study performed in Brittany, genotyping was successful in two out of the four patients colonized by *P. jirovecii* (Nevez et al., 2018). A 13-week old infant had a mixed infection with the combination of genotype 2 and genotype 3, associated with internal transcribed spacer (ITS) haplotype Jf. The second patient harbored genotype 2 in the absence of ITS haplotype identification. Both patients harbored a dihydropteroate synthase (DHPS) wild type (threonine55/ proline57). In the Brazilian study, mtLSUrRNA genotyping was successful in 12 patients and also showed a predominance of genotype 1 (5/12, 41.6%) and lower frequencies of genotype 3 (3/12, 25%) and genotype 2 (2/12, 16.6%). In addition, all patients harbored a DHPS wild type. A Spanish study has analyzed the longitudinal distribution of mtLSUrRNA genotypes in CF patients. During a 1-year follow-up, a continuous infection-and-clearance cycle of genotypes was observed with a switch from genotype 1 to genotype 3 (Montes-Cano et al., 2007). The results of another Spanish study were consistent, since a predominance of genotype 3 in CF adult patients was observed (Montes-Cano et al., 2006).

### *Pneumocystis* Primary Infection in CF Infants

*Pneumocystis* primary infection is a common phenomenon in non-immunosuppressed infants without underlying diseases, with a peak of occurrence between the third and fifth months (Vargas et al., 2001; Larsen et al., 2007; Nevez et al., 2020). It occurs contemporaneously to a self-limiting infection in the course of which the pulmonary tract is colonized by the fungus (Vargas et al., 2001; Larsen et al., 2007; Nevez et al., 2020). Primary infection occurs probably in the same manner in CF infants. Consistently, in the course of the study performed in Rennes, *P. jirovecii* was detected in a 13-week old infant who recovered without specific treatment (Nevez et al., 2018). Nonetheless, two PCP cases in CF infants have been recorded in the literature. The first one concerned a 15-week old HIV-seronegative infant newly diagnosed with CF who underwent a BAL to determine pneumonia etiology. BAL examination revealed *P. jirovecii* asci and pathogens usually associated with CF, such as *S. aureus* and *P. aeruginosa*. The patient recovered after cotrimoxazole treatment (Royce and Blumberg, 2000). The second one concerned a 16-week old HIV-seronegative infant newly diagnosed with CF who underwent a BAL to investigate chronic cough etiology. BAL examination revealed *P. jirovecii* asci and *P. aeruginosa*. The patient recovered after cotrimoxazole treatment (Kaur et al., 2016). The young age of the two infants strongly suggested that they effectively developed *Pneumocystis* primary infection. These two case-reports showed that PCP contemporaneous to severe primary infection occurs in CF infants in the apparent absence of risk factors other than the genetic disease itself.

## *Pneumocystis* Pneumonia in CF Patients After Lung Transplantation

Beyond pulmonary colonization, overt PCP occurs in CF patients after lung transplantation due to immunosuppressive therapy. In Spain, in the course of a 14-year follow-up, 57 CF patients underwent lung transplantation. Surprisingly none developed PCP (Solé et al., 2006). In Italy, in the course of a 6-year follow-up, 55 CF patients underwent lung transplantation. Three patients developed PCP (5.4%), two of whom died (Quattrucci et al., 2005). In Poland, in the course of a 7-year follow-up, 21 CF patients underwent lung transplantation. Eight patients (38%) developed PCP (Wojarski et al., 2018). Thus, the risk of PCP in CF patients after lung transplantation appears highly variable.

## General Discussion

There are several hypotheses to explain differences in prevalence of pulmonary colonization with *P. jirovecii* in European and Brazilian CF patients.

Differences may result from technical issues. In the European studies, available specimens were sputa whereas in the Brazilian study it was BALs which provided high-quality cells from the alveolus and consequently a higher sensitivity to *P. jirovecii* detection (Pederiva et al., 2012). A qPCR assay was used in the Brittany study (Nevez et al., 2018) instead of two-step PCR assays which were performed in the other studies (Sing et al., 2001; Respaldiza et al., 2005; Hernandez-Hernandez et al., 2012; Pederiva et al., 2012; Green et al., 2016). Sputum examination or the qPCR assay might have been less sensitive than BAL examination and the two-step-PCR assays.

Climatic parameters may affect *P. jirovecii* presence in CF patient populations and may explain geographical variations of prevalence. The factor of a Mediterranean climate in Seville and a temperate, maritime climate in Brittany has already been discussed (Le Gal et al., 2010; Nevez et al., 2018). Unfortunately, information was mainly obtained from Europe and Brazil, which limited the discussion on putative relationship between geographical location and *P. jirovecii* incidence in CF patients. Nonetheless, geographical variations could be non-independent factors (Miller et al., 2007, 2018). Indeed, population densities in European regions that differ from each other may affect inter-individual encounters and consequently *P. jirovecii* transmission and acquisition.

A history of cotrimoxazole treatment for bacterial infections may play a role in *P. jirovecii* clearance from the lungs, thus influencing the results of *P. jirovecii* detection. For instance, two out of 88 patients in Seville compared to 30 out of 76 patients in Brest had a history of cotrimoxazole treatment (2.2 vs. 39.4% *p* < 0.001) (Respaldiza et al., 2005; Le Gal et al., 2010). Furthermore, in Manchester, *P. jirovecii* was more frequent in patients not treated with cotrimoxazole (29.7 vs. 0%, *p* = 0.03) (Green, 2015).

Attention should be paid to other patients' characteristics, such as CFTR gene mutations. Two of the three patients in the Rennes study who tested positive for *P. jirovecii* were heterozygous for the F508Del mutation (Nevez et al., 2018). Nonetheless, considering the lack of information in the other studies a putative relationship between the presence of *P. jirovecii* and CFTR mutations remains speculative, requiring further evaluation.

The presence of *P. jirovecii*, its relationship with patients' age, pulmonary microbiota and the severity of CF must be debated. Bacterial infections arise earlier than fungal infections during the disease's progression, both acting as aggravating factors in respiratory dysfunction (Caverly et al., 2019; Delhaes et al., 2019; Cuthbertson et al., 2020). CF patients are initially infected with *H. influenzae* and *S. aureus*, and further develop infections with *P. aeruginosa* or *Burkholderia cepacia* complex. The use of antibiotics to prevent or treat infections by these bacteria, has resulted in improving the clinical outcome and increasing the life expectancy of CF patients (Castellani et al., 2018). Consequently, they are at risk for mycoses (Schwarz et al., 2018) but not for *P. jirovecii* infections, according to our analysis. The median ages of the patients enrolled in the Spanish (15.8 years) and Brazilian (11 years) studies (Respaldiza et al., 2005; Pederiva et al., 2012) were lower than those of the patients enrolled in the French multicenter study (23.5 years) (Hernandez-Hernandez et al., 2012), the Brittany study (19 years) (Nevez et al., 2018), and the British study (mean age, 31 years) (Green et al., 2016). The colonization with *P. jirovecii* may be transient and may occur earlier during CF progression, which is consistent with the presence of *P. jirovecii* in younger Spanish and Brazilian patients and the scarcity of *P. jirovecii* detection in older Breton and British patients.

*In vivo* competitive inhibition between species and networks between fungal and bacterial kingdoms in CF may exist (Soret et al., 2020). For instance, *P. aeruginosa* may limit the spread of *P. jirovecii* within the lungs. Indeed, in the French multicenter study, the patients colonized by *P. aeruginosa* mucoid strains were less likely to be colonized by *P. jirovecii* (Hernandez-Hernandez et al., 2012). Moreover, *P. jirovecii* colonization was associated with less severe lung disease according to FEV1 values, which appeared consistent with the negative correlation between *P. jirovecii* and *P. aeruginosa* mucoid strains. On the other hand, *P. jirovecii* colonization was associated with *P. aeruginosa* colonization in Brazilian patients (Pederiva et al., 2012). However, it remains unknown whether these patients were specifically infected with mucoid strains. and the relationship between *P. jirovecii* and mucoid strains is only one of the possible hypotheses.

*P. jirovecii*'s role as an aggravating factor on its own is unclear in CF patients, while it has been previously suggested in patients with COPD (Morris and Norris, 2012). The Manchester study may be consistent with this role in CF patients since the presence of *P. jirovecii* was significantly associated with exacerbation (Green et al., 2016), in contrast to the French multicenter study (Hernandez-Hernandez et al., 2012), in which the presence of *P. jirovecii* was associated with less severe lung diseases. We can hypothesize that *P. jirovecii* provokes a local inflammation facilitating subsequent infections such as by *P. aeruginosa*, in the course of which the fungus could be detectable or not (Ulrich et al., 2010; Döring et al., 2011; Hernandez-Hernandez et al., 2012; Pederiva et al., 2012). Finally, whether the fungus's presence is the cause or the result of exacerbation remains an open question.

Most data on microbiota resulted from routine culture and identification. Today, NGS methods allow identifying a higher number of microbial species, and thus to analyze cooperative, competitive and/or adaptive interactions between microorganisms that play a role in CF disease progression (Quinn et al., 2014; Layeghifard et al., 2019; Soret et al., 2020). However, these methods are mainly based on amplification of ITS sequences to analyze mycobiota, *i.e.*, fungal microorganisms. These sequences are present in multicopies in most fungal genomes but are unfortunately present in a single copy in the *P. jirovecii* genome (Nahimana et al., 2000). Thus, despite their potential efficiency, NGS methods may not be sensitive enough to capture *P. jirovecii* sequences present in relative average/low abundance within the mycobiota profiles (Delhaes et al., 2012).

Analyses of the results of *P. jirovecii* genotyping from cross-sectional studies must be made cautiously. Indeed, a continuous colonization/clearance cycle with changes of mtLSUrRNA genotype prevalence, e.g., with a switch of genotype 1 to genotype 3 has been identified (Montes-Cano et al., 2007). Other studies not focusing on CF patients suggest that single-nucleotide polymorphism (SNP) at position 85 is somehow correlated to fungal burden levels and virulence. A putative link between SNP 85T or 85A, high fungal burdens and infection severity has been suggested whereas SNP 85C may be related to low/average burdens and less severe infections (Esteves et al., 2010a,b, 2016). The switch from genotype 1 (85C/248C) to genotype 3 (85T/248C) in CF patients who, nevertheless, do not develop severe infections remains unclear. Genotype 3 may be the most adapted in the course of CF disease progression. It is unfortunate that, in the Manchester study which highlighted significant association of *P. jirovecii* and exacerbation, genotyping was not performed. No DHPS mutant-types were identified in patients from Brittany or Brazil, consistent with the general low prevalence of mutant types previously reported in these two locations (Wissmann et al., 2006; Le Gal et al., 2013a). In the Brittany study, a 13-week old infant at risk for *Pneumocystis* primary infection harbored a combination of mtLSUrRNA genotype 2 and genotype 3, associated with ITS haplotype Jf and a DHPS wild type (Nevez et al., 2018). No additional data on genotypes in a context of primary infection in CF infants are available. Indeed, the two case reports on severe primary infection that we mentioned above did not provide any information about genotypes (Royce and Blumberg, 2000; Kaur et al., 2016). Conversely, data exist on genotypes in non-CF infants developing primary infection (Totet et al., 2003a,b; Nevez et al., 2020). In Brittany, *P. jirovecii* genotyping was performed in non-immunosuppressed infants developing symptomatic primary infection. MtLSUrRNA genotype 2 was the most frequent (13/21, 61, 9%) whereas, none harbored genotype 1. Thus, the presence of genotype 2 in the aforementioned CF infant may reflect particular geographical characteristics of the fungus in infants living in this region. ITS haplotype Jf has rarely been described in France, whatever the patient population (range, 0–7.5%), whereas Eg is the most frequent haplotype (range, 42.8–64.5%) (Nevez, 2003; Le Gal et al., 2013b, 2015b). The presence of type Jf in this infant could simply be a random event. Finally, all these genotypic characteristics were obtained using a unilocus approach and Sanger's method of sequencing, while more informative assays such as multilocus sequence typing (MLST) combined with NGS are available today (Alanio et al., 2016; Charpentier et al., 2017). Moreover, shotgun metagenomics may represent an alternative method to identify pulmonary mycobiota including *P. jirovecii* organisms (Irinyi et al., 2020).

The detection of *P. jirovecii* does not seem to be predictive of subsequent PCP occurrence since no colonized patients enrolled in the above-mentioned studies developed PCP. In the absence of profound immunodeficiency, the shift from colonization to PCP did not occur. In lung transplant recipients with iatrogenic immunodeficiency, attack rates of PCP have been assessed at 5–5.8% or 6.5–43% in patients with or without chemoprophylaxis, respectively (review in reference Iriart et al., 2015). However, precise data on attack rates of PCP among transplanted CF patients were not provided in this review. The differences in PCP occurrence in CF patients from Spain, Italy, and Poland (Quattrucci et al., 2005; Solé et al., 2006; Wojarski et al., 2018) may be explained by different uses of PCP chemoprophylaxis.

Although rare, PCP can also occur contemporaneously to *Pneumocystis* primary infection in CF infants (Royce and Blumberg, 2000; Kaur et al., 2016) and the medical community should be aware of this. Early impairment of pulmonary function due to the genetic disease, in *P. jirovecii* immune-naïve infants, in the apparent absence of immunosuppression, could be the cause of PCP occurrence. Indeed, this early impairment is objectivized by the presence of *S. aureus* and *P. aeruginosa* in infants a few weeks old, while these bacteria are usually detected in older patients with pulmonary dysfunction and more advanced disease. Conversely, this hypothesis is not consistent with the negative association between mucoid *P. aeruginosa* and *P. jirovecii* suggested in the French multicenter study (Hernandez-Hernandez et al., 2012). Nevertheless, microorganism interactions in CF patients including infants are certainly complex. Moreover, whether PCP prophylaxis in CF infants is required remains an open question.

Finally, data on *P. jirovecii* and CF are fragmented. This topic remains an open field of investigation. A multicenter prospective study comprising identical methods of *P. jirovecii* screening in CF patients including those who have undergone lung-transplantation, with patient-age stratification, CFTR mutation analysis, and MLST/NGS analysis of the *P. jirovecii* genome combined with microbiome/mycobiome examination is warranted.

## Author Contributions

PB and GN: wrote, corrected, and submitted the manuscript. SL: wrote, corrected, and provided biological data from CF patients in Brest. EC: wrote the paragraphs on genomic diversity. LD: wrote paragraphs on microbiota. DQ: performed biological diagnoses and provided biological data from CF patients in Brest. FR-G: read and corrected the manuscript, wrote the reference section, and provided biological data from Rennes. SR: provided clinical data from CF patients and represents the Réseau Muco-Ouest. All authors contributed to the article and approved the submitted version.

## Conflict of Interest

The authors declare that the research was conducted in the absence of any commercial or financial relationships that could be construed as a potential conflict of interest.
